# Secondary Prevention Strategies for Ischemic Stroke in Antiphospholipid Syndrome

**DOI:** 10.3390/jcm14228026

**Published:** 2025-11-12

**Authors:** Jonathan Naftali, Sheree Finkelshtain, Eitan Auriel

**Affiliations:** 1 Department of Neurology, Rabin Medical Center, Zeev Jabotinsky St. 39, Petah Tikva 4941492, Israel; 2 Faculty of Medicine, Tel Aviv University, Tel-Aviv 69978, Israel

**Keywords:** antiphospholipid syndrome, ischemic stroke, secondary prevention, vitamin K antagonists, antiplatelet therapy, statins

## Abstract

**Introduction**: Antiphospholipid syndrome (APS) is an autoimmune prothrombotic disorder associated with both venous and arterial thrombosis, most notably ischemic stroke. Patients face a high risk of recurrence, and yet optimal strategies for secondary prevention remain uncertain. **Methods**: We conducted a narrative review of the literature on secondary prevention of ischemic stroke in APS. We performed a comprehensive literature search of PubMed for English-language articles on secondary stroke prevention in APS. Studies were included if they were original human research (e.g., randomized trials, cohort, or case–control studies) or relevant reviews addressing APS-related stroke prevention. **Results**: Vitamin K antagonists (VKAs) remain the standard of care for high-risk patients with arterial events. Several randomized controlled trials demonstrated higher recurrence rates, particularly of stroke, among APS patients treated with direct oral anticoagulants (DOACs). The optimal target INR remains debated; pooled analyses suggest no clear advantage of high-intensity anticoagulation (INR 3–4) over standard-intensity (INR 2–3), but individualized adjustment is warranted in select cases. In patients with recurrence despite adequate anticoagulation, adding an antiplatelet agent may be beneficial, although supporting evidence is limited. Adjunctive statin therapy shows promise in reducing endothelial dysfunction and prothrombotic markers, with observational data suggesting a possible protective effect, although randomized evidence is lacking. In addition, patent foramen ovale (PFO) closure has been proposed in selected APS patients with paradoxical embolisms, particularly when combined with anticoagulation. Non-pharmacological strategies, including structured lifestyle modification and rigorous vascular risk-factor management, are strongly recommended, as traditional cardiovascular risk factors synergistically increase recurrence risk. **Conclusions**: Secondary prevention of ischemic stroke in APS requires an individualized approach. VKAs remain first-line, with consideration of antiplatelet add-on, statins, lifestyle interventions, and PFO closure in appropriate settings. Future well-designed clinical trials are needed to refine INR targets, validate combination strategies, and clarify the role of adjunctive therapies in this complex patient population.

## 1. Introduction

Antiphospholipid syndrome (APS) is a systemic autoimmune disorder defined by the persistent presence of antiphospholipid antibodies (aPLs), including lupus anticoagulant (LA), anticardiolipin (aCL), and anti-β2-glycoprotein I (anti-β2GPI) antibodies [1,2]. These antibodies are directly implicated in the pathogenesis of a hypercoagulable state, leading to both arterial and venous thrombotic events, as well as pregnancy-related complications such as recurrent miscarriage and preeclampsia [3].

Neurological manifestations, particularly ischemic stroke [4,5], are among the most severe and debilitating complications of APS, representing a leading cause of long-term morbidity.

Given the high risk of recurrent thrombotic events in APS patients, effective secondary prevention strategies are crucial to reduce morbidity and mortality. This review aims to synthesize current evidence on secondary prevention strategies for ischemic stroke in APS, focusing on pharmacological and non-pharmacological interventions, risk stratification, and future research directions.

To our knowledge, no previous reviews have comprehensively synthesized both antithrombotic regimens and adjunctive measures for secondary stroke prevention in APS; we aim to provide that integrated, clinician-focused perspective.

## 2. Methods

This article is a narrative review aimed at synthesizing current evidence regarding secondary prevention of ischemic stroke in patients with APS.

### 2.1. Search Strategy

We conducted a targeted literature search in PubMed using both medical subject headings (MeSH) and free-text keywords to identify studies addressing secondary prevention of ischemic stroke in patients with antiphospholipid syndrome (APS). The search combined the following MeSH terms and related keywords: “Antiphospholipid Syndrome,” “antiphospholipid antibodies,” “anticardiolipin,” “anti-β2 glycoprotein I,” “lupus anticoagulant,” “ischemic stroke,” “cerebral infarction,” “transient ischemic attack,” “recurrent stroke,” “secondary prevention,” “anticoagulants,” “anticoagulation,” “warfarin,” “vitamin K antagonists,” “direct oral anticoagulants,” “DOACs,” “NOACs,” “rivaroxaban,” “apixaban,” “dabigatran,” “heparin,” “low molecular weight heparin,” “antiplatelet therapy,” “aspirin,” “clopidogrel,” “dual antiplatelet therapy,” “statins,” “hydroxychloroquine,” “blood pressure control,” “lipid lowering,” “smoking cessation,” and “lifestyle modification”. Boolean operators (AND/OR) were used to combine search terms, and MeSH terms were exploded where applicable. Additional relevant articles were identified through manual review of reference lists from key publications. Guideline recommendations were extracted from major international societies [6,7,8,9]. 

Sources included human studies focused on secondary prevention of ischemic stroke in APS, encompassing randomized controlled trials, cohort, and case–control studies, systematic reviews, meta-analyses, and expert consensus guidelines. The search was limited to human studies published in English, without restriction on publication year. Titles and abstracts were screened for relevance (J.N. and S.F.), and full texts of potentially eligible studies were reviewed.

### 2.2. Data Extraction and Quality Assessment

Given the narrative scope of this review, no formal risk-of-bias assessment or quantitative synthesis was performed. Instead, data on study design, population, interventions, and outcomes were extracted and synthesized qualitatively. Priority was given to higher-quality evidence and studies with direct relevance to APS-related stroke prevention.

## 3. Results

### 3.1. Direct Oral Anticoagulants (DOACS) vs. Vitamin K Antagonists (VKAS)

#### 3.1.1. Thrombosis and Bleeding Occurrence

Few RCTs evaluated the efficacy of anticoagulation. RAPS [10] RCT (2016) compared rivaroxaban to warfarin in patients with APS. The primary endpoint was percentage change in endogenous thrombin potential (ETP), and the study focused on patients with a prior VTE (not necessarily high-risk). The study showed non-inferior thrombin potential and no clinical thrombosis in either arm for 210 days [10]. Among high-risk triple-positive APS patients, the multicenter TRAPS RCT (Rivaroxaban vs. warfarin; n = 120; mean follow-up 569 days) was terminated early due to an increased rate of recurrent thrombosis events in the rivaroxaban arm (11/59 (19%)) versus the warfarin arm (2/61 (3%)) [11]. Another noninferiority RCT with 190 patients over 3 years confirmed these findings: recurrent thrombosis occurred in 11.6% rivaroxaban vs. 6.3% VKA users, with AIS seen only in the DOAC group [12]. Yet, the ASTRO-APS trial (apixaban vs. warfarin) was halted prematurely after observing 6/23 (26%) strokes in the apixaban group and none in the VKA arm [12]. On the other hand, in the real-world cohort study comparing apixaban versus VKAs in APS patients, apixaban was associated with a lower but statistically nonsignificant risk of recurrent thromboembolism (6.1% vs. 14%; HR 0.33; 95% CI, 0.10–1.04) [13] (see Table 1).

A recent meta-analysis showed that patients who were randomized to DOACs showed a significantly higher risk of arterial thrombosis compared to those on VKAs (10.3% vs. 1.3%; OR 5.43, 95% CI 1.87–15.75; *p* < 0.001), driven largely by stroke events—while the composite of arterial or venous thrombosis was also elevated (11.5% vs. 2.5%; OR 4.46, 95% CI 1.12–17.84; *p* = 0.03) [15].

Across randomized controlled trials, no significant differences in major bleeding rates were found between DOACs and VKAs. In the TRAPS trial, major bleeding occurred in 7% of DOAC and 3% of VKA patients [11], while Ordi-Ros [14] reported rates of 6.3% and 7.4% major bleeding, respectively [14]. Meta-analysis confirmed these findings, showing similar rates of major bleeding between groups (4.3% vs. 4.2%; OR 1.02; *p* = 0.97) [15].

#### 3.1.2. INR Levels Target

Pooled data from retrospective studies and RCTs [16,17,18,19] showed no difference in thrombosis recurrences between treatment with VKA with a target INR of 2–3 and of 3-4 (relative risk (RR) 0.46 (0.06–3.52) [6]. However, these studies included a mixture of patients with either venous or arterial thrombosis, and only a minority had arterial events. In the one trial that provided data specifically on patients with arterial thrombosis, there was no difference in recurrences between those treated to a target INR of 2–3 or an INR of 3–4 but without statistical significance (HR 3.1 (0.3–30.0)), although the sample was small and the achievement of a target INR of 3–4 was low [16].

#### 3.1.3. Guidelines

Reflecting these findings, as well as other meta-analyses [15,20,21], guidelines now advise against DOAC use in high-risk APS. Both EULAR [6] and ISTH [7] guidelines recommend VKAs as first-line therapy, particularly for APS patients with arterial events or triple antibody positivity. As for the INR target, both suggest that treatment with VKA with INR 2–3 or INR 3–4 can be used, considering the individual’s bleeding/thrombosis risk. However, there is no guidance on the management of recurrent arterial events under treatment.

### 3.2. Antiplatelet Therapy

#### 3.2.1. Thrombosis and Bleeding Occurrence

Limited RCTs exist on the efficacy of antiplatelet therapy in APS patients. The only available RCT [22] in Japan evaluated secondary prevention after ischemic stroke and compared aspirin alone with aspirin plus warfarin (target INR 2.0 to 3.0; mean 2.4 ± 0.3; n = 20). Primary endpoint: recurrent ischemic stroke over a mean of 3.9 years. Recurrence was higher with aspirin alone (*p* = 0.026), though the number of events in the aspirin arm was not reported.

Observational cohorts suggest that antiplatelet-based strategies, either alone or combined with VKAs, may achieve outcomes comparable to VKAs. Using two databases (a single-center APS clinic dataset and a multicenter APS registry), a multicenter retrospective cohort [23] of secondary prevention after arterial thrombosis (n = 139) found VKA plus aspirin 6.9% vs. VKA 23.75% vs. Single antiplatelet therapy (SAPT) 37.2% for any recurrent thrombosis (arterial n = 18, venous n = 10, and microvascular n = 1) over a median of 4.24 years. INR 2 to 3 was used in the single-center dataset; intensity was not recorded in the registry. Therapy was continued as long-term secondary prevention. A single-center retrospective cohort in Japan [24] (secondary prevention after arterial events; n = 90) compared warfarin n = 13, SAPT n = 41, warfarin plus antiplatelet n = 21, and dual antiplatelet therapy (DAPT) n = 15. The endpoints were time to recurrent thrombosis and time to major bleeding or death. Inclusion required at least two years of prophylaxis; median follow-up was 8 years overall. DAPT duration was not protocol-defined. Where warfarin was used, intensity was moderate (PT INR about 1.5 to 2.5 at recurrence). Recurrence per 100 patient years: warfarin was 11.6, SAPT was 5.5, warfarin plus antiplatelet was 3.7, and DAPT was 1.8. A retrospective cohort in Korea [25] of aPL or APS-related ischemic stroke or TIA (n = 167) modeled exposure as time varying across warfarin, SAPT, DAPT, and DOAC. Primary endpoint: composite of recurrent thrombosis and major bleeding from first secondary preventive prescription to last follow-up, with separate component analyses. SAPT and DAPT reduced the composite vs. warfarin, with DAPT showing lower major bleeding; recurrent thrombosis was similar across groups. Median follow-up up was about 2.5 years in the antiplatelet groups (about 0.5 years in the DOAC group). Warfarin INR was not reported. There was no prespecified DAPT duration.

In a network meta-analysis [26] of 13 studies, 719 patients (follow-up 0.1 to 20 years) showed warfarin plus an antiplatelet reduced recurrent overall thrombosis vs. SAPT (RR 0.41). DAPT ranked best for preventing recurrent arterial events, but without statistical significance (RR 0.29). DOACs increased recurrent arterial events (RR 4.06). Across the included VKA arms, INR targets were mostly 2 to 3 (overall range 1.5 to 4.0) (see Table 2).

#### 3.2.2. Guidelines

International guidance for secondary prevention after ischemic stroke in APS is not uniform. EULAR 2019 supports low-dose aspirin 75 to 100 mg daily for asymptomatic aPL carriers with a high risk profile, such as, for example, triple positivity [6]. BSH 2024 does not recommend routine primary prophylaxis with aspirin in asymptomatic carriers and favors an individualized risk-based approach [27].

For secondary prevention after APS-related ischemic stroke, VKAs are first-line therapy with a target INR of 2 to 3 [6]. In selected high-risk arterial APS or in recurrent events, adding low-dose aspirin to a VKA may be considered, and some experts escalate the INR target to 3 to 4 according to bleeding and recurrence risk [6]. Antiplatelet therapy alone is reserved for patients with a contraindication to anticoagulation or for those who clearly have a lower thrombotic risk [6]. Current guidelines do not recommend DAPT as a substitute for VKA because evidence is limited and largely non-randomized [6,27].

### 3.3. Statin Therapy

In a multicenter retrospective cohort of thrombotic APS [28] (n = 184; mean follow-up of 48.5 months), statin use was associated with fewer recurrent events, with adjusted hazard ratios of 0.24 to 0.28 and an IPTW adjusted hazard ratio of 0.28. In an open-label prospective study [29] of antiphospholipid antibody-positive patients, three months of fluvastatin 40 mg daily significantly and reversibly reduced six of twelve proinflammatory and prothrombotic biomarkers that were elevated at baseline, but clinical outcomes were not assessed.

For expert guidance, the review by Garcia and Erkan [1] summarizes plausible mechanistic and clinical rationale for statin use. It notes that while statins reduce antiphospholipid antibody-induced endothelial activation and prothrombotic biomarkers, randomized APS trials are still lacking. Statins are considered a potential adjunct, especially in patients with concurrent cardiovascular risk.

### 3.4. Patent Foramen Ovale (PFO) Closure

Evidence for PFO closure in antiphospholipid syndrome is limited and comes mainly from observational studies in broader thrombophilia populations. In a retrospective cohort of 136 patients with thrombophilia, 31 percent (n = 42) had antiphospholipid syndrome; PFO closure was associated with a five-fold reduction in recurrent stroke or TIA compared with medical therapy (*p* = 0.02) [30]. Other studies demonstrated a hazard ratio of 0.25 (95% CI, 0.08–0.74; *p* = 0.012) favoring closure over medical therapy in 134 patients with thrombophilia, though specific APS data were not provided [31]. In a large multicenter cohort of patients who underwent closure after systematic thrombophilia screening, the recurrence rate was about 0.8 per 100 person years, new-onset atrial fibrillation occurred about 1.0 per 100 person years, and in-hospital procedural complications were 3.1 percent, with no signal of higher risk among patients with thrombophilia [32]. For comparison, in a medically treated cohort with PFO and or an interatrial septal aneurysm, the recurrence rate was 8.4 percent over follow-up, approximately 2.1 percent per year [33].

#### Guidelines

International guidance on PFO closure for secondary prevention in APS is not uniform. AHA and ASA 2021 and ESO 2024 consider closure reasonable in carefully selected adults aged 18 to 60 with embolic-appearing nonlacunar ischemic stroke, no alternative cause, and high-risk PFO features, typically with long-term antiplatelet therapy [8,9]. When lifelong anticoagulation is indicated for APS, the incremental benefit of adding closure over anticoagulation alone is uncertain and should be individualized with shared decision-making [34]. SCAI 2022 suggests that closure, in addition to lifelong anticoagulation, may be considered in selected thrombophilia cases, whereas EULAR 2019 focuses on vitamin K antagonist therapy and does not specifically endorse PFO closure [6,35].

### 3.5. Lifestyle Modifications

Direct evidence for lifestyle modifications in secondary stroke prevention among APS patients remains notably limited. A systematic search identified no studies specifically examining lifestyle interventions for secondary stroke prevention in confirmed aPL-positive patients. However, observational data suggest that traditional cardiovascular risk factors significantly influence thrombotic risk in this population. One study found that hypertension and smoking were associated with arterial thrombotic events in 77 APS patients, with the combination of risk factors further increasing arterial thrombosis risk [36]. Another study demonstrated that hypertension, hyperlipidemia, and tobacco use increased arterial thrombosis risk in 222 aPL-positive patients, recommending smoking cessation and cholesterol lowering as potential disease-modifying interventions, though without quantifying their direct effect on recurrence [37]. A recent study reported a five-fold increased risk of recurrent thrombosis associated with obesity (*p* = 0.01) in a cohort of 98 APS patients that was followed for 9 years [38].

### 3.6. Risk Stratification

The risk of recurrent ischemic stroke in APS varies by antiphospholipid antibody profile.

In a retrospective analysis of primary APS, triple-positive status was strongly associated with recurrent thrombotic events (odds ratios 6.06–20.71), and IgG anticardiolipin and IgG anti-β2 glycoprotein I carried odds ratios of 10.33–21 and 7.5–32, respectively [39]. A separate retrospective analysis reported that triple positivity conferred more than a three-fold increase in recurrent thrombotic events [37]. Registry data from the retrospective APSantiCO cohort documented a 44 percent ten-year recurrence of thrombotic events among triple-positive patients [40]. In a cross-sectional and retrospective study of 70 APS patients, the presence of lupus anticoagulant predicted recurrent thrombotic events with an odds ratio of 7.3 (95% CI 1.19–45.1) [41].

#### Guidelines

Current guidance recommends risk stratification by the antiphospholipid antibody profile. Consensus statements and guidelines advise that patients with a high-risk profile—in particular, those who are triple-positive or lupus anticoagulant-positive—are at the highest risk of recurrence. For such patients, and for those with arterial events, including ischemic stroke, vitamin K antagonists are recommended as a first-line therapy, and direct oral anticoagulants are discouraged; when vitamin K antagonists are used, an INR target of 2 to 3 or 3 to 4 may be selected based on bleeding versus thrombosis risk [6]. The 2023 ACR EULAR classification criteria reinforce this stratification framework by formally weighting triple positivity and lupus anticoagulant in the classification of APS, thereby aligning classification with the high-risk biological profile used in management decisions.

## 4. Discussion

This review synthesizes current evidence regarding secondary prevention strategies in patients with APS who experienced AIS. Although APS-related stroke is an important clinical concern, primary APS accounts for only a small fraction of all acute ischemic strokes [42], limiting the available evidence. The findings highlight important challenges in therapeutic decision-making, particularly concerning the role of INR target, antiplatelet add-on, adjunctive therapies such as statins, and non-pharmacological interventions, including lifestyle modification and structural interventions.

Evidence from randomized and observational studies consistently suggests that VKAs are the cornerstone of primary, as well as secondary, prevention in APS patients with arterial events [6,20,21], especially those with triple antibody positivity [6]. As shown, multiple RCTs demonstrated an excess risk of recurrent arterial thrombosis, predominantly ischemic stroke, in patients treated with DOACs compared with VKAs.

The optimal intensity of VKA therapy remains uncertain. While earlier studies explored whether higher INR targets (3–4) might reduce recurrences compared to standard-intensity (2–3), pooled evidence suggests no significant difference. However, most available studies were underpowered, included heterogeneous patient populations, and reported limited data specific to arterial events. Thus, while current guidelines permit either INR target, individualized decision-making based on thrombotic and bleeding risk is warranted. Therefore, following an ischemic event, is it reasonable to target INR between 3 and 4.

In clinical practice, a significant challenge arises when patients with APS experience unstable INR values, poor adherence, or intolerance to VKAs. In such cases, several strategies can be adopted. First, and most importantly, optimization of anticoagulation management through patient education, closer INR monitoring, and the use of dedicated anticoagulation clinics can help achieve more stable therapeutic ranges. Second, in patients with persistently fluctuating or subtherapeutic INR despite optimal management, the addition of low-dose aspirin to standard-intensity VKA may be suggested. Third, long-term use of low molecular weight heparin (LMWH) or switching to a different VKA agent may be considered in selected cases, especially in those with contraindications to VKAs or during periods of labile INR control.

The role of antiplatelet therapy in secondary prevention of AIS is less well-defined. Observational data suggest that combination regimens—such as VKA plus SAPT or DAPT—may further reduce recurrence compared to VKA alone. Nonetheless, these findings are largely retrospective and lack validation in prospective randomized settings. Consequently, we support adding SAPT to VKAs for secondary prevention in high-risk patients with labile INR.

Beyond anticoagulation and antiplatelets, statins emerge as promising adjuncts. Retrospective cohorts suggest a protective effect of statins against recurrent thrombosis, possibly by mitigating endothelial dysfunction and prothrombotic activation induced by antiphospholipid antibodies. Although randomized trials are lacking, their established cardiovascular benefit and favorable safety profile support their use, particularly in patients with coexisting vascular risk factors.

Lifestyle modification remains a cornerstone of secondary prevention of AIS, regardless of etiology. Control of hypertension, hyperlipidemia, and smoking cessation significantly lowers thrombotic risk, and adopting a Mediterranean diet or engaging in regular physical activity may offer additional benefits.

Furthermore, as APS predisposes patients to deep vein thrombosis, paradoxical embolism through a PFO should be considered in patients with recurrence of cortical stroke. The retrospective studies of PFO closure in patients with thrombophilia, along with low complication risk, are in favor of PFO closure in patients with AIS on VKAs, as well as continuing VKAs therapy.

Lastly, it is essential to perform a comprehensive work-up in patients with recurrent ischemic stroke to exclude alternative or concomitant mechanisms beyond APS. This should include malignancy screening when clinically indicated (e.g., elevated D-dimer or suggestive imaging/clinical findings) [43], prolonged cardiac rhythm monitoring such as Holter or event recorders to detect occult atrial fibrillation [44], and echocardiographic evaluation to identify potential embolic sources.

Clinicians may apply the evidence summarized here to tailor prevention strategies according to antibody profile, recurrence history, and comorbid vascular risk—bridging current rheumatologic and neurologic practice.

This narrative review has several important limitations. First, it was not conducted as a formal systematic review, and no structured risk-of-bias assessment of the included studies was performed. Second, the literature search was limited to English-language publications, which may have excluded relevant data from non-English sources and introduced selection bias. Third, much of the available evidence derives from small observational cohorts, post hoc analyses, or prematurely terminated trials, contributing to heterogeneity across studies and limiting the robustness of conclusions. Fourth, some of the data summarized in this review originated from both primary studies and systematic reviews that included those same studies, leading to potential duplication and overemphasis of certain findings. This overlap may distort the perceived weight of evidence in favor of specific therapeutic strategies. Finally, there remains a paucity of high-quality randomized trials to guide optimal INR targets, combination regimens, and the role of adjunctive measures such as statins or PFO closure, particularly in patients with recurrent arterial events. These factors collectively limit the generalizability and certainty of our conclusions.

### Future Directions

Addressing the gaps in APS-related stroke prevention will require targeted research efforts. Firstly, well-designed multicenter trials are needed to determine optimal antithrombotic strategies—for example, defining the ideal INR intensity for VKAs or the role of combination therapy (VKAs plus antiplatelets) in patients with recurrent events. Given the low prevalence of primary APS among stroke patients, international collaboration and patient registries will be essential to achieve adequate sample sizes and statistical power in future studies. Finally, future research should stratify outcomes by patient subgroups—for instance, examining differences by sex, age, or aPL antibody profile—to inform a more personalized approach to secondary stroke prevention in APS.

## 5. Conclusions

Secondary prevention of AIS in APS requires an individualized approach. First, a thorough diagnostic work-up to exclude alternative etiologies should be performed. VKAs remain the standard of care for high-risk patients, while the addition of an antiplatelet agent and close INR monitoring should be considered in cases of recurrence. Adjunctive statin therapy, together with structured non-pharmacological interventions and comprehensive vascular risk factor management, is an essential component of optimal care. Ultimately, ongoing research and well-designed clinical trials are needed to refine secondary prevention strategies and improve outcomes in this complex patient population.

## Figures and Tables

**Table 1:** Randomized controlled trials of DOACs vs. VKAs in APS.

Study	Drug (DOAC)	Population and N	Follow-Up	Thrombosis Recurrence	Major Bleeding
Moore et al. (RAPS), 2016 [10]	Rivaroxaban	VTE-APS, n = 116	210 days	0% both	0% both
Pengo et al., TRAPS, 2018 [11]	Rivaroxaban (20/15 mg)	Triple-positive APS, n = 120	569 days	19% vs. 3% (HR ≈ 6.7; increase risk in rivaroxaban patients for arterial strokes/MI)	7% vs. 3%
Ordi-Ros et al., 2019 [14]	Rivaroxaban (20/15 mg)	APS, n = 190	3 years	11.6% vs. 6.3% (RR ≈ 1.83; strokes only DOAC)	6.3% vs. 7.4%
Woller et al., ASTRO-APS, 2021 [12]	Apixaban (2.5 or mg BID)	APS, n = 48	12 months	6 events vs. 0	0 vs. 1

**Table 2:** Key clinical evidence for antiplatelet therapy.

Study and Design	Therapy	Population	Follow-Up	Thrombosis Recurrence	Major Bleeding
Okuma et al., 2009: Double-blind RCT [22]	Aspirin 100 mg vs. Aspirin + Warfarin (INR 2.0–3.0)	APS with prior stroke (n = 20)	3.9 years	Higher recurrence in aspirin-only group	Similar between groups
Jackson et al., 2017: Retrospective cohort [23]	SAPT vs. VKA vs. VKA + ASP	APS with arterial thrombosis (n = 139)	4.24 years	37.2% (SAPT) vs. 23.7% (VKA) vs. 6.9% (Combo)	Not reported
Ohnishi et al., 2019: Retrospective cohort [24]	SAPT, DAPT, VKA (1.5–2.5), VKA + SAPT	APS with arterial thrombosis (n = 90)	8 years	1.8 (DAPT), 3.7 (Combo), 5.5 (SAPT), 11.6 (VKA)/100 PY	No significant difference
Yang et al., 2025: Retrospective cohort [25]	SAPT, DAPT, Warfarin, DOAC	aPL or APS with prior ischemic stroke or TIA (n = 167)	2.5 years in all groups except the DOAC group (0.5 year)	Composite outcome HR 0.24 (SAPT), HR 0.25 (DAPT) vs. Warfarin	
Attachaipanich et al., 2023: Network meta-analysis [26]	SAPT, DAPT, VKA, VKA + SAPT, DOAC	APS with arterial thrombosis (13 studies, n = 719)	Varies	RR 0.41 (VKA + SAPT), RR 0.29 (DAPT) vs. SAPT	No significant difference

## Data Availability

No new data were created or analyzed in this study.
